# An Investigation into the Growth of *Lolium perenne* L. and Soil Properties Following Soil Amendment with Phosphorus-Saturated Bauxite Residue

**DOI:** 10.1007/s00128-022-03514-6

**Published:** 2022-04-07

**Authors:** Patricia B. Cusack, Mark G. Healy, Oisín Callery, Elisa Di Carlo, Éva Ujaczki, Ronan Courtney

**Affiliations:** 1 Department of Biological Sciences, University of Limerick, Castletroy, Co. Limerick Ireland; 2 The Bernal Institute, University of Limerick, Castletroy, Co. Limerick Ireland; 3 Civil Engineering and Ryan Institute, National University of Ireland, Galway, Ireland; 4 Earth and Ocean Sciences and Ryan Institute, National University of Ireland, Galway, Ireland

**Keywords:** Phosphorus, Wastewater, Phosphorus recycling, Bauxite residue, Fertiliser replacement

## Abstract

Reuse options for bauxite residue include treatment of phosphorus (P)-enriched wastewaters where the P-saturated media offers fertiliser potential. However, few studies have assessed the impact on soil properties. Two types of spent P-saturated bauxite residue were applied to soil and compared to conventional superphosphate fertiliser as well as a control soil. Soil physico-chemical properties, worm *Eisenia fetida* L. choice tests, and *Lolium perenne* L. growth and elemental uptake were examined. Comparable biomass and plant content for *L. perenne* in the P-saturated bauxite residue treatments and those receiving superphosphate, indicated no phytotoxic effects. *E. fetida* L. showed a significant preference for the control soil (58 %± 2.1%) over the amended soils, indicating some form of salt stress. Overall, P-saturated bauxite residue was comparable to the superphosphate fertiliser in terms of the plant performance and soil properties, indicating the potential recycling of P from wastewaters using bauxite residue as a low-cost adsorbent.

Bauxite residue, the by-product from the alumina industry, is produced at an annual global rate of 150 Mt. Current re-use rate of bauxite residue is estimated at < 15%, the remainder of which is being disposed of into bauxite residue disposal areas (BRDAs) (Ujaczki et al. 2018; Chao et al. 2022). There is therefore urgent need to determine suitable reuse options for residue and several studies have assessed its potential for removing phosphorus (P) from waste streams (Grace et al. 2016; Cusack et al. 2018). The recovery and reuse of phosphorus (P) is viewed as a central tenet of a circular bioeconomy (Jarvie et al. 2019), and can help solve the P paradox (Baker et al. 2015). However, relatively few studies have evaluated this reuse potential (Arenas-Montaño et al. 2021) and no study has investigated the re-use of spent P-saturated bauxite residue. The potential application of saturated media to land as an alternative P source to inorganic fertiliser may be limited due to limited P desorption, elevated metal content and/ or inherent properties of the waste material. Additionally and due to the alkaline, sodic and saline nature of bauxite residue, potential stress responses to both plant and soil fauna require evaluation when considering the application of the P-saturated bauxite residue to land (Fourrier et al. 2020).

Therefore, the aims of this study were to (1) compare the potential of P-saturated bauxite residue, following use in water and wastewater treatment, as a fertiliser by comparing with conventional superphosphate fertiliser in terms of the growth of perennial ryegrass (*Lolium perenne* L.) (2) investigate any changes in soil chemical properties following residue addition (3) assess the impact of land application on soil fauna using *Eisenia fetida* L. choice tests (4) identify any phytotoxic effects on the germination of seeds and root growth, and (5) assess potential trace element uptake in the plant biomass from the growth media.

## Materials and Methods

A P deficient mineral soil (Morgan’s P of 0.64 ± 0.08 mg L^−1^) was selected as the control soil (Lufa-Speyer, Germany). Phosphate applications to the control soil were in the form of two types of P-saturated and chemically modified bauxite residue, and superphosphate fertiliser. The two bauxite residues originated from filters used for the removal of dissolved reactive phosphorus (DRP) in dairy soiled water (Cusack et al. 2018), and comprised two configurations: bauxite amended with 8% gypsum (BRG) or with a chemical to enhance permeability during filtration (sodium alginate) (BRC) (Cusack et al. 2018). After full P saturation was reached and the columns were deconstructed, both media were oven-dried at 105°C for 24 h, pulverised using a mortar and pestle, and sieved to a particle size < 2 mm. The superphosphate fertiliser had a P content of 16%.

Application of the media to the soil was at rates equivalent to 30 t P ha^−1^. The four treatments were: (1) a low P content control soil (Ct) (2) Ct with P-saturated bauxite residue/gypsum media (BRG) (3) Ct with P-saturated chemically-modified bauxite residue (BRC), and (4) Ct with superphosphate fertiliser (SP).

Soil pH and electrical conductivity (EC) of the treatments were measured in aqueous extract at a 1:5 ratio (solid: liquid) (Courtney and Harrington 2010). The elemental composition (Na, Ca, K, Mg, Cu, Fe, Mn, Zn and Al) of the bauxite residue, fertiliser and control soil used in this study was determined by ICP following microwave digestion.

Water extractable P was determined using dried and sieved (< 2 mm) soil, 1 g in 20 mL deionised water was shaken for 1 h at 180 rpm on a reciprocal shaker. Morgan’s P analysis was carried out using 0.54 M CH_3_COOH and 0.7 M NaCH_3_COO at a pH 4.8. Olsen P analysis was conducted using 0.5 M NaHCO_3_ at pH 8.5 (Olsen 1954). Samples were analysed by spectrophotometry following digestion for total P (TP) and total nitrogen (TN) content (HACH DR3900, APHA 4500-N and APHA 4500-P).

Earthworm (*E. fetida* L) bioassays such as choice (avoidance) tests allow for the easy identification of any avoidance behaviour exhibited by worm and are commonly used in soil ecotoxicological tests to indicate potential stress induced by elevated contaminant content (Udovic and Lestan 2010).

The avoidance behaviour of the earthworm *Eisinea fetida* L. (locally sourced) was examined using a six-sectioned preference chamber (n = 3) in accordance with ISO 17512-2 (2007). Each of six-interconnected chambered stainless steel avoidance ring segments were filled with approximately 700 cm^3^ of soil and the corresponding treatment applied. Twenty-five *E. fetida* L. individuals were placed into the central cavity of each ring, and earthworms present in each chamber recorded after 72 h.

Seed germination and root elongation tests for *Lolium perenne* L. were carried out using water extracts and seedling performance was assessed using the relative seed germination (RSG) after Courtney and Mullen (2009).

The RHIZOtest™ procedure (ISO 16198:2015) was used to assess element phytoavailability in the amended soils. The method was based on modifications reported by Di Carlo et al. (2020), who used the Rhizotest to assess trace element uptake in bauxite residue. In brief, the trial consisted of two main phases: a growing (or hydroponic) phase, where *Lolium perenne* L. seeds were germinated and the seedlings grown in an aerated nutrient solutions over 10 days; and an exposure (or contact) phase, when the planar root mat (grown on a polyamide mesh) was placed in contact with the test soils (n = 5 per treatment) over 14 days. The treatments BRG, BRC and SP were immersed in a P-deficient solution containing 2000 µmol dm^−3^ KNO_3_, 2000 µmol dm^−3^ Ca(NO_3_)_2_ and 1000 µmol dm^−3^ MgSO_4_. After the 14 days’ exposure, the plant biomass was harvested and oven-dried at 60°C for 72 h. The dry weight (DW) plant biomass was determined and then digested in a microwave system according to CEN/TS 15,290:2006 before elements analysis (Al, Ca, Mg, K, Na, Cu, Fe and Zn) by ICP-OES (Thermo Fisher iCAP 7400 Radial). Plant reference material (from LGC Promochem, UK) was also prepared with recovery rates of 91%–100% determined.

Differences between soil properties and plant growth parameters in the different treatments was performed using Tukey's post hoc tests on one-way ANOVA using SPSS Version 21.

## Results and Discussion

The elemental composition of the bauxite residue (Table 1) was consistent with previous descriptions (Gräfe et al. 2011), which also measured high contents of Fe, Al, Ca and Na. Superphosphate was also high in Ca, with appreciable Zn content.

**Table 1:** Total element composition (mg/kg) of the bauxite residue and control soil used studied

	Bauxite Residue	Control Soil	Fertiliser
Na	23,000	164	3700
Ca	39,000	1700	199,600
K	575	362	2400
Mg	855	585	1550
Cu	7	6	20
Fe	308,900	3600	560
Mn	209	150	7
Zn	67.5	18	250
Al	61,900	4800	400

**Fig. 1:**
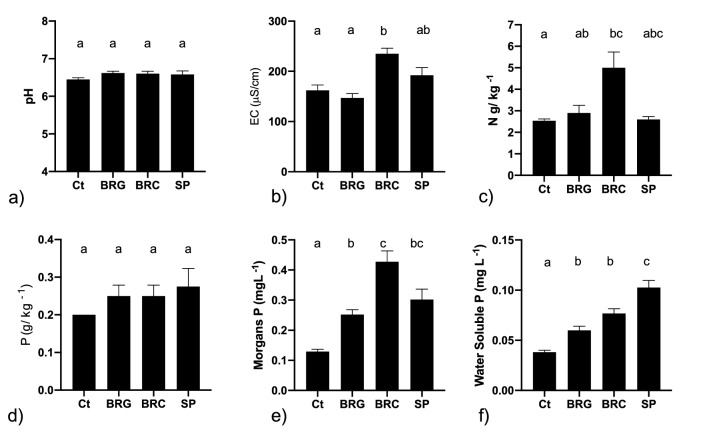
Selected soil parameters following application of P-saturated media; **a** pH, **b** electrical conductivity (EC), **c** total N, **d** total P, **e** Morgans P, and **f** water soluble P. Values plotted are mean ± SEM and bars sharing the same letter indicate no significant difference at the *p* < 0.05 level using one-way analysis of variance (ANOVA) followed by Tukey’s multiple comparison test

There was no significant difference (*p* > 0.05) measured between soil pH values and treatments (Fig. 1a). The pH ranged from 6.4 ± 0.1 to 6.62 ± 0.11 and fit in the desirable pH range for plant growth, which is between pH 5.5 and 9 (Mendez and Maier 2007). An 8-week pot trial study carried out by Summers et al. (2000), who applied bauxite residue-coated superphosphate fertiliser at a rate of 20 t ha^−1^ found that there was an increase in pH from 3.9 to 6.2. Similarly, Ruyters et al. (2011) found that the pH of soil increased from 6.8 to 8.3 after 3 weeks of plant growth following the addition of bauxite residue at a rate of 16.5%. However, at similar application rates, Fourrier et al. (2020) found that while bauxite residue increased soil pH by about 1 unit, there was no increase when modified with gypsum residue applied at same rate.

A significant difference was detected in the salinity, as measured by the EC, between the control and the soil receiving the gypsum-treated bauxite residue (Fig. 1b). Significant differences were also noted in the salinity between the gypsum-treated bauxite residue (BRG) and the BRC treatment. However, optimal plant growth requires soil EC of < 4000 µS cm^−1^ and values measured for all treatments in this study was below this value. No significant difference (*p* > 0.05) was detected in soil TP between the treatments (Fig. 1d). However, there was a significant difference (*p* < 0.05) in the TN content of the study control and the treatments (Fig. 1c).

Bauxite residue is often an undesirable growth media for plants due to its generally high alkalinity, sodicity and salinity (Courtney et al. 2009; Fourrier et al. 2020). However, many studies have highlighted the positive effects of treatments such as gypsum in the improvement of the physico-chemical properties of bauxite residue (Courtney and Mullen 2009; Fourrier et al. 2020) and, in particular, their ability to mitigate P loss from soils and increase biomass growth. For example, bauxite residue was added at a rate of 40 t ha^−1^ to a sandy soil prone to P loss and, consequently, an increase in production of 24% was noted as a result of its P retention capacity (Summers et al. 1996).

Soil test phosphorus (STP) measurements such as Morgan’s and Olsen are used to give an estimated value for soil P available for vegetative growth (Neyroud and Lischer 2003). There were significant differences in the Morgan’s P content between the control and all three treatments (Fig. 1e). Morgan’s extractable P varied from 0.13 in the control to 0.43 mg P L^−1^ in the soil receiving the chemically modified bauxite residue. This emphasises the importance of building up and managing soil P in very P-deficient soils such as those used in this study.

There was a significant difference (*p* < 0.05) between the water extractable P in the control compared to all three treatments. Water extractable P varied from within the four treatments, with the lowest amount observed for the control (0.04 mg P L^−1^) and the highest observed for the soil treated with the superphosphate fertiliser (0.10 mg P L^−1^) (Fig. 1f). Olsen (CaCl_2_) extractable P was below detection limits (< 0.02 mg L^−1^) in all soils.

Significant difference between the *E. fetida* L.’s choice of soils and the treatment applied was found (Table 2). The largest percentage (58%) of *E. fetida* L. favoured the control soil. *E. fetida* L. were distributed amongst the other treatments, with the lowest population of *E. fetida* L. (12.2%) found in the soil containing the superphosphate application, which may suggest a sensitivity to the chemical composition of the superphosphate fertiliser. Similar to the current study, Rastetter et al. (2017) and Rastetter and Gerhardt (2017) found earthworm avoidance > 80% when phosphate containing recyclates were added to soil, with conventional phosphate fertiliser eliciting the highest response. This was attributed to its high water solubility and presence of elevated metal content. Responses in the current study are most likely due to the elements associated with the P fertiliser, as amended bauxite residue has compared favourably to control soils in other studies (Finnegan et al. 2018).

**Table 2:** Percentage number of *E. fetida* L. recovered from each treatment sample at the end of the test period

	Percentage worms
Ct	57.7 ± 1.2a
BRG	14.8 ± 3.0b
BRC	15.3 ± 4.3b
SP	12.2 ± 1.6b

Means (n = 5 ± SE) followed by the same letter are not significantly different at p ≤ 0.05

**Fig. 2:**
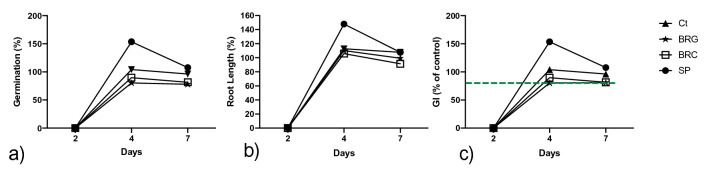
**a** Relative seed germination (RSG) percentages of four bauxite residue extracts and one control using the species *L. perenne* L*.* **b** Relative root growth (RRG) percentages of four bauxite residue extracts and one control using the species *L. perenne* L*.* **c** Germination indices (GI) percentages of four bauxite residue extracts and one control using the species *L. perenne* L*.* Broken green line indicates phytotoxic threshold

*Lolium perenne* L. germinated in all treatments (Fig. 2a), with a RSG > 73.1% observed. The rate of germination is a factor in the establishment of vegetative growth and is reduced by environmental conditions such as a highly saline growth media (Courtney and Mullen 2009). No significant relationship was detected between soil EC and the final root length. GI percentages after 7 days were $\ge$ 80% (Fig. 2c), indicating absence of phytotoxicity.

**Fig. 3:**
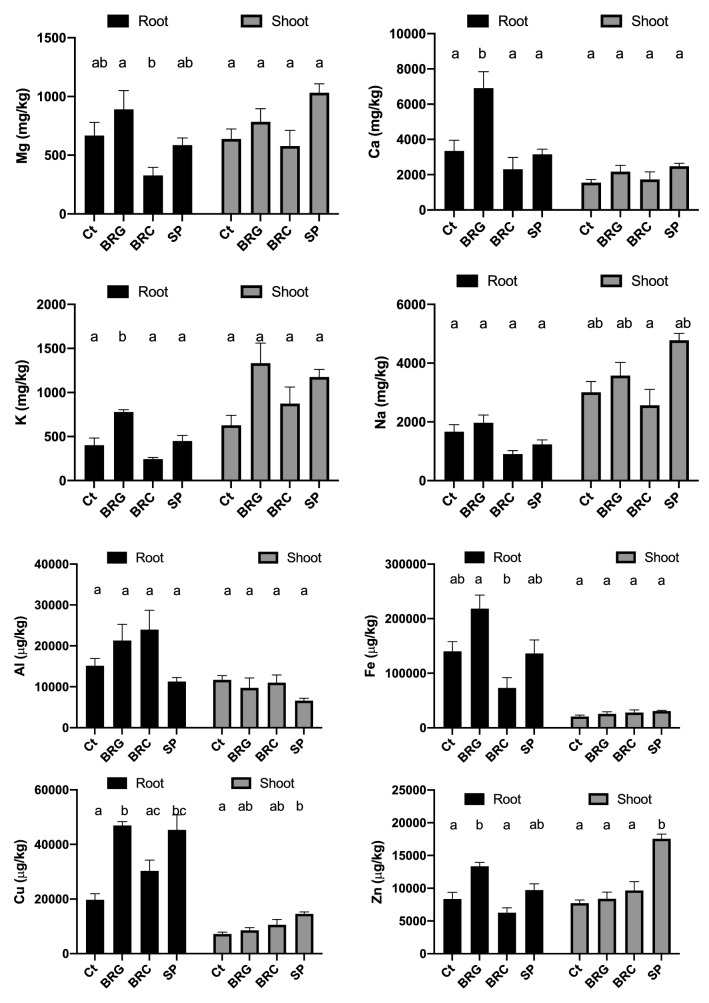
**a** Nutrient cation content in *L. perenne* root and shoot samples following Rhizotest exposure testing, **b** trace element content in *L. perenne* root and shoot samples following Rhizotest exposure testing. Values plotted are mean ± SEM and bars sharing the same letter indicate no significant difference at the *p* < 0.05 level using one-way analysis of variance (ANOVA) followed by Tukey’s multiple comparison

No significant (*p* > 0.05) differences in the plant biomass was registered among the treatments at the end of the exposure period (i.e. validity criteria met). Application of bauxite residue to soil and/or elevated Na content can suppress nutrient uptake in plants (Rutyers et al. 2011; Di Carlo et al. 2020). Of the nutrient cations, Ca was present in the highest amount with significantly higher (*p* < 0.05) concentrations in the BRG root samples (Fig. 3a). This is most likely due to the higher Ca content in the amended soil resulting from gypsum amendment. The trend of higher Ca content in the root than shoot was found for all treatments with no significant differences between shoot content.

Sodium concentration above 1% is considered a general threshold for phytotoxicity (Ruyters et al. 2011). No increased Na content was found in treatments containing the bauxite residue media (*p* > 0.05) compared to the control soil (Fig. 3a). Indeed, the highest shoot Na content was found in the SP treatment and reflects the higher content of Na in SP compared to the control soil (Table 1) and the solubility of SP fertiliser. Application of BRG to the soil resulted in increased K content for both root (*p* < 0.05) and shoot (*p* > 0.05) samples. Slightly lower values of K were recorded for the BRC and SP treatments. Overall, plant nutrient content was at the lower end of the range of typical concentrations for non-intensive grassland species (Ca = 0.1%–1%; K = 1%; Mg = 0.1%−1%) (Bradshaw and Chadwick 1980) and reflect the low nutrient content of the control soil. These findings suggest that the amended residue is not inhibitory to nutrient uptake in grassland and indicate the potential for saturated media from effluent treatment as a supply of other nutrients such as K.

Application of amendments such as bauxite residue and nutrient sludges to soils can lead to elevated concentrations of elements such as Cu, Fe and Zn (Rutyers et al. 2011; Rastetter et al. 2017). All treatments resulted in higher Cu shoot content, with the highest value recorded for SP (14,558 µg kg^−1^) compared to 7186 µg kg^−1^ in the control (Fig. 3b). Significantly higher Cu content was found for root content in all the treatments compared with the control. Conversely, the Zn shoot content did not increase in BRG and BRC treatments, but was higher in the SP treatment reflecting the Zn content of the fertiliser (Table 1).

Iron content for all treatments were higher in root than shoot content (Fig. 3b). In the root samples, the lowest values were recorded for BRC, indicating that amended bauxite residue does not increase Fe availability in soil. The higher values recorded for BRG were not significantly higher than those for the control soil. No significant differences were found amongst the treatments for shoot content.

Elevated root Al content was observed for BRG-amended soil treatments, but the differences were not significant (*p* > 0.05). While soil amended with bauxite residue can contain elevated aqueous Al content, significant decreases were observed when residue was gypsum-amended and is attributed to soil pH of < 8.5 (Lehoux et al. 2013). The soil pH in the current study was below this threshold value and when comparing shoot content there was no differences in Al content. Land application of P saturated modified bauxite residue media does not pose adverse effects on elemental uptake.

This study investigated the efficacy of bauxite residue as a nutrient source on the growth of *Lolium perenne* L. and its impact on soil chemical properties, and compared its impact to a conventional superphosphate fertiliser. Application of P-saturated bauxite residue, in amended or unamended form, did not present phytotoxic effects on the growth of ryegrass but earthworm choice tests showed *E. fetida* L. preferred the control soil over the soils receiving the bauxite residue and the superphosphate treatments.
